# Social Determinants of Self-Reported Health in Women and Men: Understanding the Role of Gender in Population Health

**DOI:** 10.1371/journal.pone.0034799

**Published:** 2012-04-13

**Authors:** Ahmad Reza Hosseinpoor, Jennifer Stewart Williams, Avni Amin, Islene Araujo de Carvalho, John Beard, Ties Boerma, Paul Kowal, Nirmala Naidoo, Somnath Chatterji

**Affiliations:** 1 Department of Health Statistics and Information Systems, World Health Organization, Geneva, Switzerland; 2 Research Centre for Gender Health and Ageing, Faculty of Health, University of Newcastle, Newcastle, New South Wales, Australia; 3 Department of Gender, Women and Health, World Health Organization, Geneva, Switzerland; Central Institute of Educational Technology, Canada

## Abstract

**Background:**

Women and men share similar health challenges yet women report poorer health. The study investigates the social determinants of self-reported health in women and men, and male-female differences in health.

**Methods:**

Data on 103154 men and 125728 women were analysed from 57 countries in the World Health Survey 2002–2004. Item Response Theory was used to construct a composite measure of health. Associations between health and determinants were assessed using multivariate linear regression. Blinder-Oaxaca decomposition partitioned the inequality in health between women and men into an “explained" component that arises because men and women differ in social and economic characteristics, and an “unexplained" component due to the differential effects of these characteristics. Decomposition was repeated for 18 countries in the World Health Organization (WHO) African region and 19 countries in the WHO European region.

**Results:**

Women's health was significantly lower than men's. Health was associated with education, household economic status, employment, and marital status after controlling for age. In the pooled analysis decomposition showed that 30% of the inequality was “explained", of which almost 75% came from employment, education, marital status. The differential effects of being in paid employment increased the inequality. When countries in Africa and Europe were compared, the “explained" component (31% and 39% respectively) was largely attributed to the social determinants in the African countries and to women's longevity in the European countries. Being in paid employment had a greater positive effect on the health of males in both regions.

**Conclusions:**

Ways in which age and the social determinants contribute to the poorer health status of women compared with men varies between groups of countries. This study highlights the need for action to address social structures, institutional discrimination and harmful gender norms and roles that differently influence health with ageing.

## Introduction

Protecting and promoting the health of women and men is not only a basic human right, it is also crucial for health and economic development in all nations. It is important to ensure that health systems are responsive to women's and men's needs yet this requires a robust evidence base [1], [2]. Data collection on health outcomes must take into account the cultural, social, economic and systemic determinants of health for women and men as they age. Moreover, appropriate methodologies are necessary for the analysis of data to inform policies aimed at improving health [3]. Additionally there is a need for clarity in the terminology. In doing equity analysis, inequality and equality refer to measurable quantities, whereas inequity and equity are value-based concepts.

The purpose of this paper is to explore the social determinants of health in men and in women and to explain male-female differences in self-reported health. The study advances understanding of men's and women's health and how gender affects the health of women. Gender refers to the different socially constructed roles, norms, behaviours, activities, and attributes that a given society considers appropriate for men and women. In many societies, these different social constructions privilege men over women producing gender inequalities, which disproportionately affect the health of women [4].

This study is important for a number of reasons. Firstly, the data derive from a large multi-country data set comprising information uniformly collected at the individual level across high, middle and low-income countries. Secondly, a rigorous statistical technique is used to ensure comparability in the health measure across populations. Thirdly, the focus is specifically on the social determinants of health in women and men, and fourthly, a decomposition method shows how social factors contribute to our measured health difference between women and men.

## Methods

The aims of this study are to: identify and describe how social factors separately determine health in adult males and females; measure and evaluate the effects of sex (that is, being female or male) on health, after adjusting for the effects of age and the social determinants; and decompose the extent to which social and other factors explain male-female differences in health status. In addition, the study explores the differential effects of social determinants on health in two geographical regions.

### Sample and data collection

The World Health Survey (WHS) was conducted by the WHO to provide representative and comparable population data on the health status of adults, aged 18 years and older, in 70 countries from all regions of the world [5] http://www.who.int/healthinfo/survey/en/index.html. All country samples were probabilistically selected but in China, Comoros, the Republic of the Congo, Côte d'Ivoire, India, and the Russian Federation, the WHS was carried out in geographically limited regions. To adjust for the population distribution represented by the United Nations Statistical Division (http://unstats.un.org/unsd/default.htm) and also non-response, post-stratification corrections were made to sampling weights [6], [7].

The study sample comprises 57 countries participating in the WHS. Inclusion at the country level required complete information on sampling weights, health status descriptions and the covariates of interest. Among the 13 excluded countries, 11 did not have data on sampling weights and two did not have the information required to calculate household wealth. In the final un-weighted sample, 55% were women, 28% were aged 50 years or above, 32% had less than primary education, 67.0% were married or cohabiting, 45% were unemployed (or not working for pay) and 49% resided in rural areas.

A comparison of health inequalities between two WHO regions was also undertaken to show how social determinants contribute to health inequalities in two distinct geographic and economic regions. (See also http://www.who.int/about/regions/en/index.html for more background). The study comprised 18 countries from the African region and 19 countries from the European region.

### Dependent variable

The health status measure derives from 16 WHS self-reported questions grouped into eight health domains: vision, mobility, self-care, cognition, interpersonal activities, pain and discomfort, sleep and energy, and affect. The Item Response Theory (IRT) partial credit model [8] was used to construct a composite measure of health status at a multi-country level. The score obtained from the model was transformed to a scale ranging from 0 (worse health status) to 100 (best health status) [7], [9].

### Independent variables

In addition to sex, the independent variables (all categorical) were: participants' age (expressed categorically as 18–19, 20–29, 30–39, 40–49, 50–59, 60–69 and 70+ years); marital status (married/cohabiting vs. never married vs. divorced/separated/widowed); educational level (no education/incomplete primary vs. complete primary vs. secondary/high school vs. college completed or above); employment status (not employed vs. employed); area of residence (rural vs. urban), and country of residence. A dichotomous hierarchical ordered probit model was used to develop an index of household economic status based on owning selected assets and/or with access to certain services [10], [11], [12]. The index was divided into quintiles within each country. The selection of independent variables was consistent with the findings of the Commission on Social Determinants of Health [13].

### Analysis

The final sample comprised 251257 respondents, from which 22375 records were removed from the dataset because of missing data on one or more variables. Two pooled datasets of 103154 males and 125728 females were analysed. (Table S1 shows each country's final sample by sex). Initial data screening and profiling involved estimating mean health status scores for the independent variables by males and females separately.

The multivariate linear regression comprised two steps. In the first, the male and female data sets were analysed separately to test the effects of all the independent variables together on health status. In the second, the pooled male/female data (N = 228882) were analysed to assess the effect of sex on health after controlling for possible confounding variables. Interaction terms between sex and the other social determinants were included and tested in the pooled model. Although we report the pooled model without interaction terms, the model with interactions is available upon request.

Multivariate regression provides the basis for a technique, known as Blinder-Oaxaca decomposition [14], which gives additional explanatory power. The decomposition method partitions the inequality in an indicator between two groups (e.g. male-female difference in health status) into two components. This first is the “explained" component which arises because the two groups, on average, have different values for the known characteristics (i.e. the characteristics that were used as the determinants in the regression). The second component is the “unexplained" part. Decomposition attributes this to the differential effects that the characteristics have on each group as well as other factors not included in the multivariate regression model [15].

Here decomposition was firstly undertaken on the study sample of 57 countries. Secondly decompositions for the WHO African and European regions were compared in order to show possible regional differences in the roles of the social determinants. Table S1 gives the WHO region for each country in the study.

All analyses were carried out using STATA version 11 (StataCorp, 2009). The Oaxaca command in Stata [16] was used and the “pooled" option was specified. The “pooled" option uses the coefficients from a pooled model over both groups (including a group indicator) as the reference coefficients [17]. For the sake of completeness, we ran the decomposition with two other options. The first used the average of the coefficients over both sex groups as the reference coefficients [18] thereby giving the same weight to the coefficients in the male and female models, and the second involved weighting the coefficients in the male and female models by male and female group sizes respectively to establish reference coefficients [19]. The results were similar under each option. Sampling weights that took into account the selection probability of the individual were included in the analysis. This weight reflected each country's population, in such a way that if the sample size for two given countries are the same (but the population sizes of the countries are different), more weight is given to the country with higher population when calculating the pooled estimates. Allowance was made for the non-independence of observations within each survey cluster.

## Results

### All countries

Table 1 shows the sample distribution of health score means for each of the independent variables for men and women separately. Health scores decreased with increasing age for both sexes. In each age group, the mean health score for women was worse than the mean health score for men in the succeeding older decade. For instance, on average, young women aged 20 to 29 years had poorer health than men aged 30 to 39. For both men and women, married/cohabiting people had better health than those who were divorced/separated/widowed, but worse health than those who had never been married/cohabiting. Health status was positively associated with higher household economic status and higher educational levels for both men and women. Those working for pay (employed) had better health compared with those not in paid employment. Respondents living in urban areas had better health than those living in rural areas.

**Table 1:** Health status scores for men and women by selected background characteristics, pooled data of 57 countries, World Health Survey, 2002–2004.

	Men		Women	
Age	Mean	SE	Mean	SE
18–19 years	83.2	0.4	80.1	0.5
20–29 years	81.4	0.2	77.1	0.2
30–39 years	79.3	0.2	73.8	0.2
40–49 years	76.0	0.2	70.9	0.2
50–59 years	73.1	0.3	67.2	0.3
60–69 years	68.4	0.3	63.2	0.3
70+ years	63.4	0.3	59.0	0.3
**Marital status**				
Married/cohabiting	76.0	0.2	72.2	0.2
Never married	81.4	0.2	78.2	0.3
Divorced/separated/widowed	71.0	0.4	64.4	0.2
**Education**				
No/incomplete primary education	74.1	0.3	69.2	0.2
Primary completed	76.9	0.2	72.1	0.2
Secondary/High school completed	79.5	0.2	75.3	0.2
College completed or above	79.6	0.4	74.1	0.4
**Employment**				
Currently in paid employed	78.3	0.2	74.2	0.2
Not working for pay	74.1	0.3	70.9	0.2
**Household economic status**				
Lowest quintile	75.1	0.3	69.8	0.3
Second quintile	75.9	0.2	70.9	0.3
Middle quintile	77.0	0.3	71.7	0.2
Fourth quintile	78.5	0.3	73.0	0.3
Highest quintile	79.6	0.3	74.6	0.2
**Urban-rural residence**				
Rural area	77.1	0.2	71.9	0.2
Urban area	77.7	0.2	72.3	0.2

Note: The health status score is on a scale from 0 to 100, where 0 is worst health and 100 best health.

Table 2 shows the adjusted effects of the social determinants on health scores resulting from the multivariate models for males and females separately and pooled. Increasing age was significantly associated with declining health. Compared with never married people, divorced/separated/widowed men and women had significantly worse health. Men, and especially women, who were married or cohabiting had worse health than those who had never married. Having completed primary, secondary or higher education compared with having no or incomplete primary education, was significantly positively associated with health for both women and men, and being in paid employment compared with not being in paid employment, was significantly associated with better health for both women and men. Higher household economic quintiles were significantly associated with better health for men, but for women this positive association was only significant for the fourth and fifth quintile compared with the first. Area of residence was not significantly associated with the health of either men or women. In the pooled model being female had a significant negative effect on health.

**Table 2:** Multivariate Analysis of Health Status of Men and Women Separately and Pooled, by Social Determinants, pooled data of 57 countries, World Health Survey, 2002–2004.

		Men			Women			All adults		
		Coefficient*	95% CI		Coefficient*	95% CI		Coefficient*	95% CI	
**Sex** (Reference category: males)		**-**	-	-	**-**	-	-	**−3.7**	−4.0	−3.4
**Age** (Reference category: 18–19 years)	20–29 years	**−2.3**	−3.1	−1.5	**−2.6**	−3.6	−1.6	**−2.5**	−3.1	−1.9
	30–39 years	**−4.4**	−5.3	−3.5	**−5.2**	−6.2	−4.2	**−4.8**	−5.5	−4.2
	40–49 years	**−7.5**	−8.4	−6.6	**−8.1**	−9.1	−7.1	**−7.9**	−8.5	−7.2
	50–59 years	**−10.3**	−11.3	−9.3	**−11.3**	−12.4	−10.2	**−10.8**	−11.5	−10.2
	60–69 years	**−13.8**	−14.8	−12.8	**−14.4**	−15.6	−13.2	**−14.2**	−14.9	−13.4
	70+ years	**−17.8**	−18.9	−16.8	**−18.2**	−19.4	−17.1	**−18.1**	−18.8	−17.3
**Marital status** (Reference category: Never married)	Married/cohabiting	**−0.6**	−1.1	0.0	**−0.9**	−1.4	−0.4	**−0.5**	−0.9	−0.1
	Divorced/separated/widow	**−1.7**	−2.5	−0.9	**−2.6**	−3.2	−2.0	**−2.1**	−2.6	−1.6
**Education** (Reference category: No/incomplete primary education)	Primary completed	**0.9**	0.4	1.4	**1.3**	0.8	1.8	**1.2**	0.9	1.6
	Secondary/High school completed	**1.6**	1.1	2.2	**2.7**	2.1	3.3	**2.3**	1.8	2.7
	College completed or above	**2.7**	1.8	3.7	**3.0**	2.2	3.8	**3.0**	2.3	3.7
**Employment** (Reference category: Not working for pay)		**2.6**	2.1	3.2	**0.5**	0.1	0.8	**1.6**	1.3	1.9
**Household economic status** (Reference category: Lowest quintile)	Second quintile	**0.9**	0.3	1.4	**0.3**	−0.3	0.9	**0.5**	0.1	1.0
	Middle quintile	**1.4**	0.7	2.0	**0.5**	−0.2	1.2	**0.9**	0.4	1.4
	Fourth quintile	**2.3**	1.7	3.0	**1.3**	0.6	2.0	**1.7**	1.3	2.2
	Highest quintile	**3.2**	2.5	3.8	**1.9**	1.2	2.7	**2.5**	1.9	3.0
**Urban-rural residence** (Reference category: Rural area)		**−0.1**	−0.6	0.4	**−0.1**	−0.7	0.5	**−0.1**	−0.6	0.4
**Constant**		**81.4**	79.2	83.5	**78.8**	76.8	80.8	**82.3**	80.6	84.1
		N = 103154			N = 125728			N = 228882		
* The coefficients are also adjusted for country of residence		R-squared 0.215			R-squared 0.261			R-squared 0.258		

The multivariate decomposition for all 57 countries (Table 3) shows how the social determinants contribute to the difference in health status between men and women. Approximately 30% of the inequality was attributed to differences in a range of factors. This is the so-called “explained" component, meaning that this resulted from differences in the characteristics of men and women. Of this “explained" component, 77% of the contribution came from social determinants. The social determinants with the largest contribution to the “explained" component were employment, education and marital status in that order. Household economic status also contributed, but to a lesser extent. The remaining 23% of the “explained" inequality was attributed to differences in the distribution of age between men and women.

**Table 3:** Decomposition of Inequality in Health Status – Contributions by Determinants, pooled data of 57 countries, World Health Survey, 2002–2004.

	Mean	SE		
**Mean health status score, men**	77.4	0.2		
**Mean health status score, women**	72.1	0.2		
**Gender difference in health status**	**5.3**	**0.2**		

	Absolute contribution	95% CI		Percentage contribution to explained/unexplained component
**Explained**	**1.6**	**1.4**	**1.8**	
Age	0.4	0.3	0.5	23.0%
Marital status	0.2	0.2	0.3	15.3%
Education	0.3	0.2	0.3	15.8%
Employment	0.7	0.5	0.8	42.4%
Household economic status	0.0	0.0	0.1	2.0%
Urban-rural residence	0.0	0.0	0.0	0.1%
Country of residence	0.0	−0.1	0.1	1.4%
**Unexplained**	**3.7**	**3.5**	**4.0**	
Age	0.0	−0.2	0.2	0.6%
Marital status	−0.1	−0.3	0.1	−2.5%
Education	−0.1	−0.2	0.1	−1.5%
Employment	0.1	0.0	0.2	2.9%
Household economic status	0.0	0.0	0.1	0.8%
Urban-rural residence	0.0	0.0	0.0	0.0%
Country of residence	0.5	0.3	0.8	13.9%
Constant	3.2	2.9	3.5	86.0%

Note: Figures may be affected by rounding.

Approximately 70% of the health status inequality resulted from differences in the effects, on men and women, of age, social determinants and factors not in the model. This is the “unexplained" component. Employment and household economic status made small but statistically significant contributions, although the impact of household economic status was minimal. The effect of employment and household economic status increased the inequality, having stronger positive effects on the health of males than females. Country of residence contributed to 14% of the “unexplained" component. However, by far the largest contribution to the “unexplained" component was from the constant term which comprised “other factors" not in the model.

### Regional comparisons: Africa and Europe

Table 4 gives the results of separate multivariate decompositions for groups of countries in the WHO African and European regions. The difference in health status between women and men was larger in the European than the African region (6.5 units vs. 3.7 units).

**Table 4:** Decomposition of Inequality in Health Status – Contributions by Determinants, pooled data of 18 WHO African countries and 19 WHO European countries, World Health Survey, 2002–2004.

	WHO African region				WHO European region			
	Mean	SE			Mean	SE		
**Health status score, men** *	78.6	0.3			76.7	0.3		
**Health status score, women** *	74.9	0.3			70.1	0.3		
**Gender difference in health status score**	**3.7**	**0.3**			**6.5**	**0.3**		

	Absolute contribution	95%CI		Percentage contribution to explained/unexplained component	Absolute contribution	95%CI		Percentage contribution to explained/unexplained component
**Explained**	**1.2**	**0.2**	**1.5**		**2.5**	**2.1**	**3.0**	
Age	0.2	0.1	0.4	22%	1.1	0.8	1.4	43%
Marital status	0.3	0.2	0.4	23%	0.2	0.1	0.3	9%
Education	0.2	0.1	0.3	15%	0.1	0.1	0.2	4%
Employment	0.4	0.3	0.6	38%	0.5	0.4	0.7	20%
Household economic status	0.0	0.0	0.1	3%	0.1	0.0	0.1	2%
Urban-rural residence	0.0	0.0	0.0	2%	0.0	0.0	0.0	0%
Country of residence	0.0	−0.1	0.1	−2%	0.6	0.3	0.8	22%
**Unexplained**	**2.5**	**2.0**	**3.0**		**4.0**	**3.4**	**4.6**	
Age	−0.2	−0.6	0.1	−9%	−0.4	−0.8	0.0	−9%
Marital status	0.1	−0.3	0.5	5%	−0.1	−0.4	0.3	−2%
Education	0.4	−0.2	1.0	16%	−0.6	−1.2	0.0	−15%
Employment	0.2	0.1	0.3	6%	0.2	0.9	0.3	5%
Household economic status	0.0	0.0	0.1	0%	0.0	0.0	0.1	1%
Urban-rural residence	0.0	−0.2	0.2	2%	−0.1	−0.4	0.2	−3%
Country of residence	−0.3	−0.7	0.1	−11%	0.7	0.2	1.2	18%
Constant	2.4	1.4	3.3	94%	4.2	3.4	5.0	105%

Note: Figures may be affected by rounding.

* Using WHO standard, age-standardized mean health scores were similar for women in the European and African regions (72.1 and 72.5 respectively) but higher for men in the European region (77.6 compared with 76.6 in the African region).

In the African region, approximately 31% of the inequality was “explained", compared with 39% in the European region. Relative contributions made by the social determinants to the “explained" component were higher in the African than the European region. For example, employment contributed 38% in the African region compared with 20% in the European region, education contributed 15% in the African region compared with 4% in the European region, and marital status contributed 23% and 9%, respectively. In the European region, age contributed 43% to the “explained" component compared with 22% in the African region.

Approximately 69% of the inequality in health status between women and men in the African region was “unexplained". Employment (working for pay) was the only determinant in the model whose differential effects were statistically significant. The effect of being in paid employment increased the inequality. In the African region, most (94%) of the “unexplained" component was attributed to the constant - other factors not included in the model.

In the European region, 61% of the inequality was “unexplained". Employment and education respectively made statistically significant positive and negative contributions to the “unexplained" component. A substantial share of the “unexplained" component in the European region was attributed to the constant.

## Discussion

This paper makes a unique contribution to the literature on the social determinants of women and men's health as well as the debate on methodologies to undertake health equity analysis. Our examination of the largest available multi-country population-based household survey of self-reported health demonstrated an inequality in the health status of men and women with women consistently having poorer health status compared to men. We show how key social determinants contribute to this inequality by the way in which they are distributed, and also by the way in which they differently impact on the health status of men and women. Internationally, social factors that are known to be associated with reporting poor health status include education, income, employment and marital status [20], [21], [22].

In both developed and developing countries women are more likely to report poorer health than men [21], [23], [24], in both younger [25], and older [26], age groups. Our analysis of WHS data showed adult women (18+ years) reported themselves as less healthy than men across all age groups. After adjusting for the effects of age and the social determinants in the pooled multivariate regression analysis, women's health status remained significantly lower than men's health status. Internationally the evidence shows that social, cultural, economic and biological factors all impact negatively and more substantially on the health of women compared with men [4]. For example, in developing countries, these influences include factors associated with contraception, pregnancy and childbirth, and also lack of autonomy in seeking and realising health care opportunities.

In this study, separate multivariate regression analyses demonstrated that the associations between the social determinants and health status differed between males and females.

Men and women who were married or cohabiting or divorced, separated or widowed, had significantly worse health than those who had never married and this was particularly true for women. Evidence shows that social change has influenced the impact of marital status and widowhood on self-reported health [27], [28] and that the influence of marital status on health varies across cultural settings [3].

Education, income and occupation are key factors that determine social position as well as access to and control over power and resources. Social position exerts a powerful influence on the type, magnitude and distribution of health in high, low and middle-income counties [29]. In this analysis of the WHS dataset, low levels of education and household wealth were associated with poor health status as was being unemployed.

The results of the decomposition when all 57 countries were pooled showed that 30% of the inequality in health status between women and men was “explained" by differential distributions of age and the social determinants. Employment was the largest single contributor to the “explained" component, this being due to the fact that a higher proportion of men than women were in paid jobs (75.5% vs. 38.5%). Analysis of WHS responses to a question in which people stated reasons for not working for pay showed that less than 4% gave “ill health" as a reason. The main reasons given by women for not having a paid job were associated with being homemakers or caring for the family, while for men the main reasons had to do with being retired, studying or unable to find a paid job. Higher education also contributed to the “explained" component resulting from the fact that more men than women had secondary level or above education (49.4% vs. 45.8%). The data on the distribution of income, employment and education reflect unequal access to resources for women relative to men. The unequal distribution of such markers on society is indicative of gender inequalities.

Being divorced, widowed or separated was associated with poor health compared with never being married, and a much higher proportion of women than men were divorced, widowed or separated (19.8% vs. 6.8%). The relatively small contribution of household economic status to the “explained" component is due in part to the similar income distribution for men and women (39.2% of men vs. 40.5% of women in the highest two income quintiles). However this may also be because the measure was calculated at household level.

The decomposition of the inequality in health status between women and men in all 57 countries showed that 70% of the inequality was attributed to differences in the effects of age and the social determinants, as well as other explanatory factors. This is the “unexplained" component. Being in a paid job compared with being unemployed, had a much larger effect on good health for men - 2.6 units (95% CI 2.1 to 3.2) - than for women - 0.5 units (95% CI 0.1 to 0.8). Research in Poland has shown that unemployed men are more likely to report poorer health than unemployed women [30]. A study conducted in Brazil showed that work was a more important determinant of health for men than for women [23].

Separate decompositions on the two regional groupings of countries in the study showed that the “explained" part of the inequality in health status between women and men was 31% and 39% respectively in the African and European regions, compared with 30% in the pooled analysis. Marital status, education and employment contributed more to the “explained" component in the African than European region.

While age was the major contributor to the explained differences in the European region, this was largely due to the fact that women in the European region live longer and are in worse-off health as seen by the differences in the health scores. Longevity was differently associated with the inequality in health status between women and men in each region; the proportion of males and females aged 60 years or above in the European region was 20% and 29% respectively compared with 9% and 10% for males and females respectively in the African region. In addition, the country of residence also played a major role in Europe in the explained component, suggesting that differential gender roles in some European countries may be driving health disparities between men and women (in these countries) that need to be further studied.

Differences in educational levels between men and women in the European region were relatively minor (88% of men vs. 85% of women with higher than primary education) but larger in the African region (31% vs. 23%). This is perhaps the reason why education plays a much more important role in explaining sex differences in health in Africa compared with Europe.

Employment was a contributor to the “explained" part of the inequality in both WHO regions, but particularly so in the African region (38% in African countries vs. 20% in European countries). Additionally, the differential effects of employment increased the inequality between women and men (that is, through a positive contribution to the “unexplained" component) in both WHO African and European country groups, thereby having a greater positive effect on the health of males.

Being employed is important both in Africa and Europe in order to produce better health outcomes. However, perhaps in Africa, where education levels are low, especially among women, and the population is relatively young compared to Europe, the role of employment becomes even more important. Employment possibly serves a more empowering role in Africa through enabling better access to health services. The new World Development Report on gender inequality in Sub-Saharan Africa [31], shows that paid employment potentially brings greater access to health and welfare benefits. While differences in the levels of male-female earnings may also partly account for the role of paid employment in explaining gender differentials in both regions, in the European region countries this effect may be partially offset by social welfare benefits that include health and income support.

Higher levels of formal education had a larger positive effect on women's health than on men's health in the European countries meaning that education contributed negatively to the inequality between women and men in this group of countries. However, the differential effects of education were not statistically significant in African countries. The constant made a substantial contribution to the “unexplained" part of the inequality in both regions, indicating that factors other than those investigated in our study influenced the inequalities in health between women and men.

The regional comparisons highlight that sex differentials in employment, marital status and education played a major role in explaining inequalities in health status between women and men in the group of African countries in the WHS. Our results highlight the fact that the feminization of ageing is a major contributor to health differentials between men and women in Europe. These findings also suggest that sex differentials in social determinants may help to explain inequalities in health between women and men in lower income countries, and population ageing may provide greater explanation of the inequalities in higher income countries. It is also important to recognise that social determinants that are important in one region may be less important in others. In tackling gender inequality as a social determinant of health inequalities, there is no “one size fits all" solution. Policy responses must account for different social, economic and demographic circumstances in countries and regions. Generating data and conducting analyses at local levels is imperative.

This study has some limitations. Firstly, although IRT health is a population independent method, it could not identify or adjust for any systematic bias between men and women that may exist [10]. The incorporation of health examinations and biomarkers within household surveys may, in future, provide ways of validating self-reported health to some extent. Secondly, participating countries were not probabilistically selected and therefore not necessarily representative of the world or of similar groups of countries (e.g. defined by geography or income). Thirdly, the “unexplained" component of the inequality suggests that there were factors that probably contributed to the difference that were either not assessed in the WHS or were not included in the present analysis. Fourthly, the actual role and position that women have in society in each of these countries is likely to vary from country to country. Additionally, factors such as employment, marital status, education and household economic status may have interacted with health outcomes but it was not possible, through the WHS, to identify whether or not this was occurring. Lastly we acknowledge that biological differences and differences in perceived health between men and women may have contributed to the differences in self-reported health shown here.

It will be important for future studies to examine issues such as social policies related to women's empowerment within countries, women's perceived social status, economic participation in the workforce, and the meaning of major life course events such as marital separation in the context of health, well-being and ageing, in order to paint a more textured picture that explains differences in the health status of men and women. Moreover the examination of biological risks and health-related events during the life course, such as childbirth, will help our understanding of the organic factors and processes that drive some of these differences.

The unequal distribution of education, occupation and income disadvantages women relative to men and these factors are markers of gender inequalities in society. By using decomposition analysis, this work shows how employment, education, marital status, household economic status, and importantly “other factors", contribute to the inequality in health between women and men at a multi-country level [29]. This underscores the need to identify and understand what these “other factors" are, and how they differentially impact on the health of women and men.

Internationally there are calls for inter-sector collaboration and public policies to make women's lives healthier by addressing gender inequalities and other the social and economic determinants of their health [1]. This includes calls to achieve the Millennium Development Goal (MDG) 3 on gender equality and women's empowerment as a goal in itself as well as a determinant of other MDGs. This study highlights the need for action to address social structures, institutional discrimination and harmful gender norms and roles that influence health equity. In particular, research is needed to help understand pathways and mechanisms through which social determinants impact on the health of women.

### Ethics Statement

Informed consent was obtained in all surveys. A standard consent form approved by the ethics review committee was read to the respondent in the respondent's language. Once the respondent agreed to participate in the survey, if the respondent was literate the form was provided to the respondent to read over and sign and was countersigned by the interviewer. If the respondent was illiterate and gave consent to participate, the interviewer confirmed this consent and signed on the form that the respondent had been read the form, had understood the study and agreed to participate. This procedure was approved by the institutional review boards.

## Supporting Information

Table S1 Study population (final unweighted sample count) by country and sex, World Health Survey, 2002–2004.
(DOC)
