# Supplementary material for: Social Determinants of Self-Reported Health in Women and Men: Understanding the Role of Gender in Population Health
Source: PLoS One. 2012 Apr 13;7(4):e34799. doi: 10.1371/journal.pone.0034799 (PMC3326052; doi:10.1371/journal.pone.0034799)
Supplement: Table S1 — Study population (final unweighted sample count) by country and sex, World Health Survey, 2002–2004. (DOC) [file pone.0034799.s001.doc]

Table S1 Study population (final unweighted sample count) by country and sex, World Health Survey, 2002–2004.

| **Country** | **WHO Region** | **Men** | **Women** | **All adults** |
| --- | --- | --- | --- | --- |
| Bangladesh | SEAR | 2,535 | 2,921 | 5,456 |
| Bosnia and Herzegovina | EUR | 431 | 586 | 1,017 |
| Brazil | AMR | 2,110 | 2,691 | 4,801 |
| Burkina Faso | AFR | 2,226 | 2,466 | 4,692 |
| Chad | AFR | 2,060 | 2,178 | 4,238 |
| China | WPR | 1,941 | 2,008 | 3,949 |
| Comoros | AFR | 444 | 247 | 691 |
| Congo | AFR | 904 | 1,043 | 1,947 |
| Cote d'Ivoire | AFR | 1,664 | 1,194 | 2,858 |
| Croatia | EUR | 386 | 559 | 945 |
| Czech Republic | EUR | 407 | 499 | 906 |
| Dominican Republic | AMR | 2,067 | 2,373 | 4,440 |
| Ecuador | AMR | 1,844 | 2,209 | 4,053 |
| Estonia | EUR | 356 | 638 | 994 |
| Ethiopia | AFR | 2,321 | 2,481 | 4,802 |
| Finland | EUR | 449 | 546 | 995 |
| France | EUR | 282 | 152 | 434 |
| Georgia | EUR | 1,116 | 1,507 | 2,623 |
| Ghana | AFR | 1,636 | 1,967 | 3,603 |
| Hungary | EUR | 281 | 302 | 583 |
| India | SEAR | 4,498 | 4,767 | 9,265 |
| Ireland | EUR | 361 | 434 | 795 |
| Israel | EUR | 327 | 424 | 751 |
| Kazakhstan | EUR | 1,537 | 2,930 | 4,467 |
| Kenya | AFR | 1,814 | 2,453 | 4,267 |
| Lao People's Democratic Republic | WPR | 1,860 | 2,049 | 3,909 |
| Latvia | EUR | 284 | 564 | 848 |
| Luxembourg | EUR | 323 | 334 | 657 |
| Malawi | AFR | 2,150 | 2,907 | 5,057 |
| Malaysia | WPR | 2,573 | 3,083 | 5,656 |
| Mali | AFR | 1,781 | 1,070 | 2,851 |
| Mauritania | AFR | 1,171 | 1,852 | 3,023 |
| Mauritius | AFR | 1,832 | 1,975 | 3,807 |
| Mexico | AMR | 16,249 | 22,078 | 38,327 |
| Morocco | EMR | 1,937 | 2,120 | 4,057 |
| Myanmar | SEAR | 2,549 | 3,331 | 5,880 |
| Namibia | AFR | 1,492 | 2,210 | 3,702 |
| Nepal | SEAR | 3,304 | 4,401 | 7,705 |
| Pakistan | EMR | 3,288 | 2,577 | 5,865 |
| Paraguay | AMR | 2,337 | 2,755 | 5,092 |
| Philippines | WPR | 4,615 | 5,354 | 9,969 |
| Portugal | EUR | 379 | 621 | 1,000 |
| Russian Federation | EUR | 1,544 | 2,753 | 4,297 |
| Senegal | AFR | 911 | 658 | 1,569 |
| Slovakia | EUR | 568 | 1,106 | 1,674 |
| South Africa | AFR | 1,033 | 1,113 | 2,146 |
| Spain | EUR | 2,585 | 3,650 | 6,235 |
| Sri Lanka | SEAR | 2,810 | 2,997 | 5,807 |
| Swaziland | AFR | 731 | 991 | 1,722 |
| Sweden | EUR | 393 | 544 | 937 |
| Tunisia | EMR | 2,289 | 2,611 | 4,900 |
| United Arab Emirates | EMR | 1,202 | 1,106 | 2,308 |
| Ukraine | EUR | 830 | 1,472 | 2,302 |
| Uruguay | AMR | 1,436 | 1,498 | 2,934 |
| Viet Nam | WPR | 1,556 | 1,890 | 3,446 |
| Zambia | AFR | 1,708 | 2,034 | 3,742 |
| Zimbabwe | AFR | 1,437 | 2,449 | 3,886 |
| **Total** |  | **103,154** | **125,728** | **228,882** |

**Table Notation**

AMR: Region of the Americas

AFR: African Region

EMR: Eastern Mediterranean Region

EUR: European Region

SEAR: South-East Asian Region

WPR: Western Pacific Region
